# Supplementary figures and images for: Dynamical system modeling to simulate donor T cell response to whole exome sequencing-derived recipient peptides: Understanding randomness in alloreactivity incidence following stem cell transplantation
Source: PLoS One. 2017 Dec 1;12(12):e0187771. doi: 10.1371/journal.pone.0187771 (PMC5711034; doi:10.1371/journal.pone.0187771)

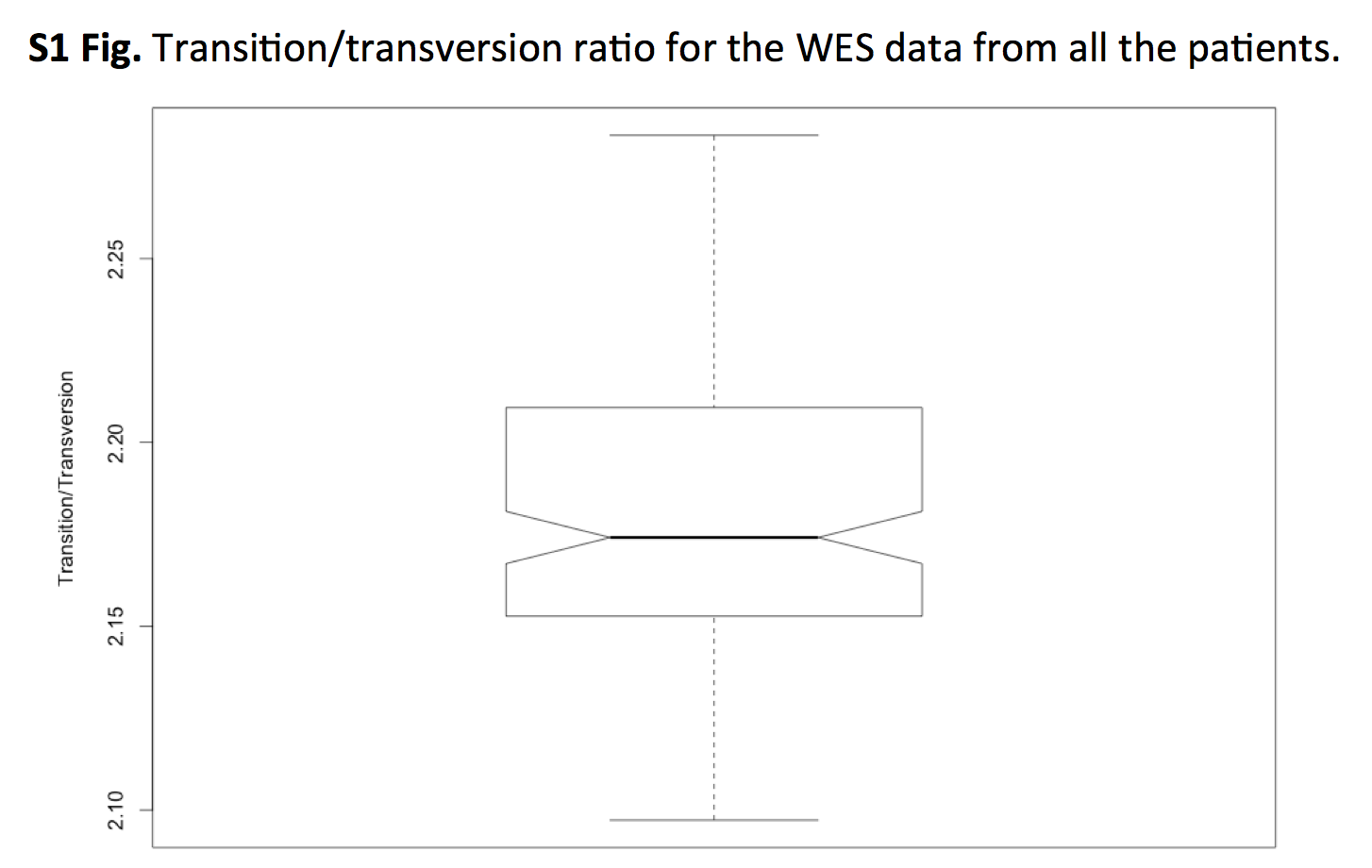

Supplement: S1 Fig — (TIFF) [file pone.0187771.s001.tiff]

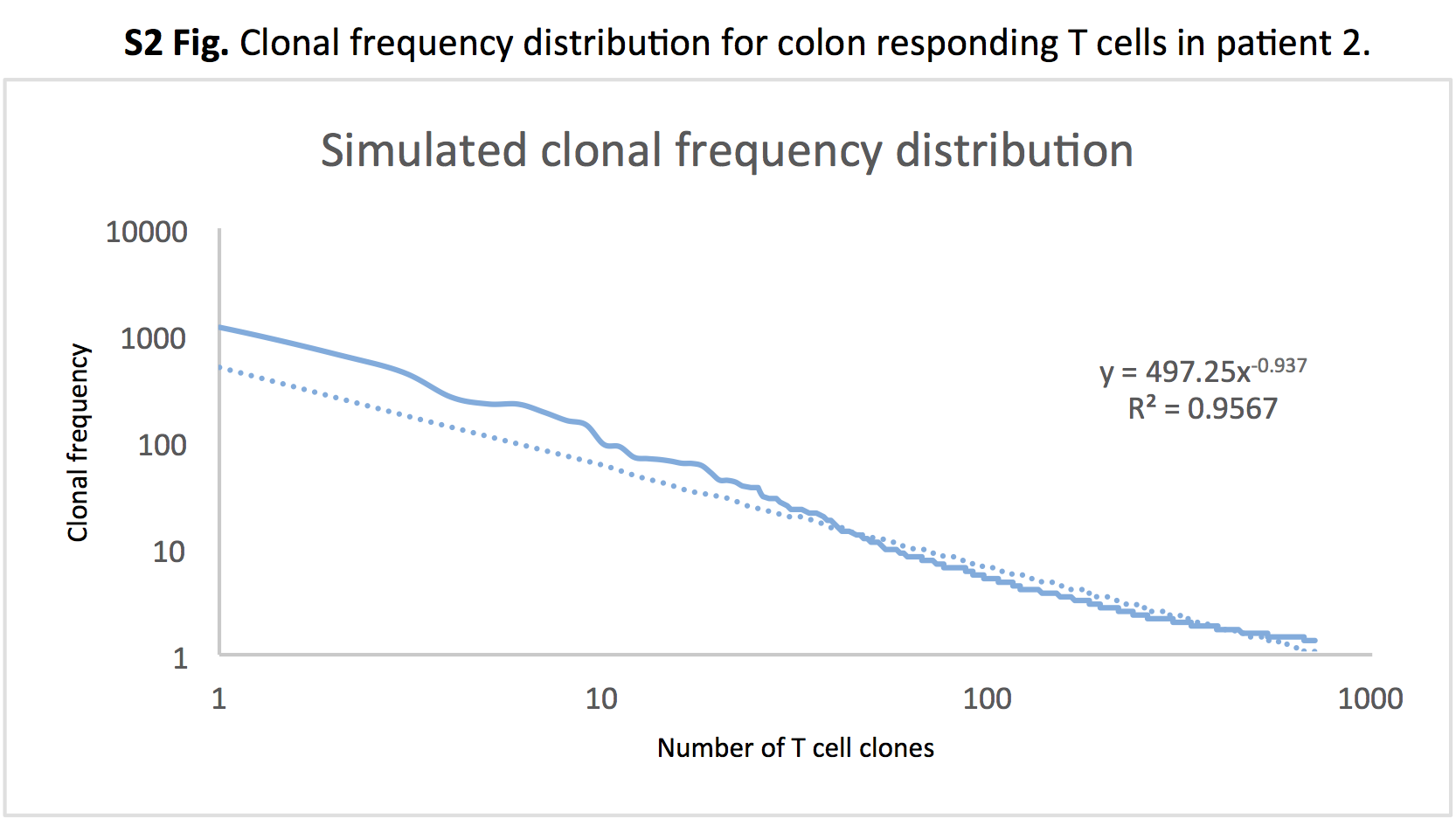

Supplement: S2 Fig — (TIFF) [file pone.0187771.s002.tiff]

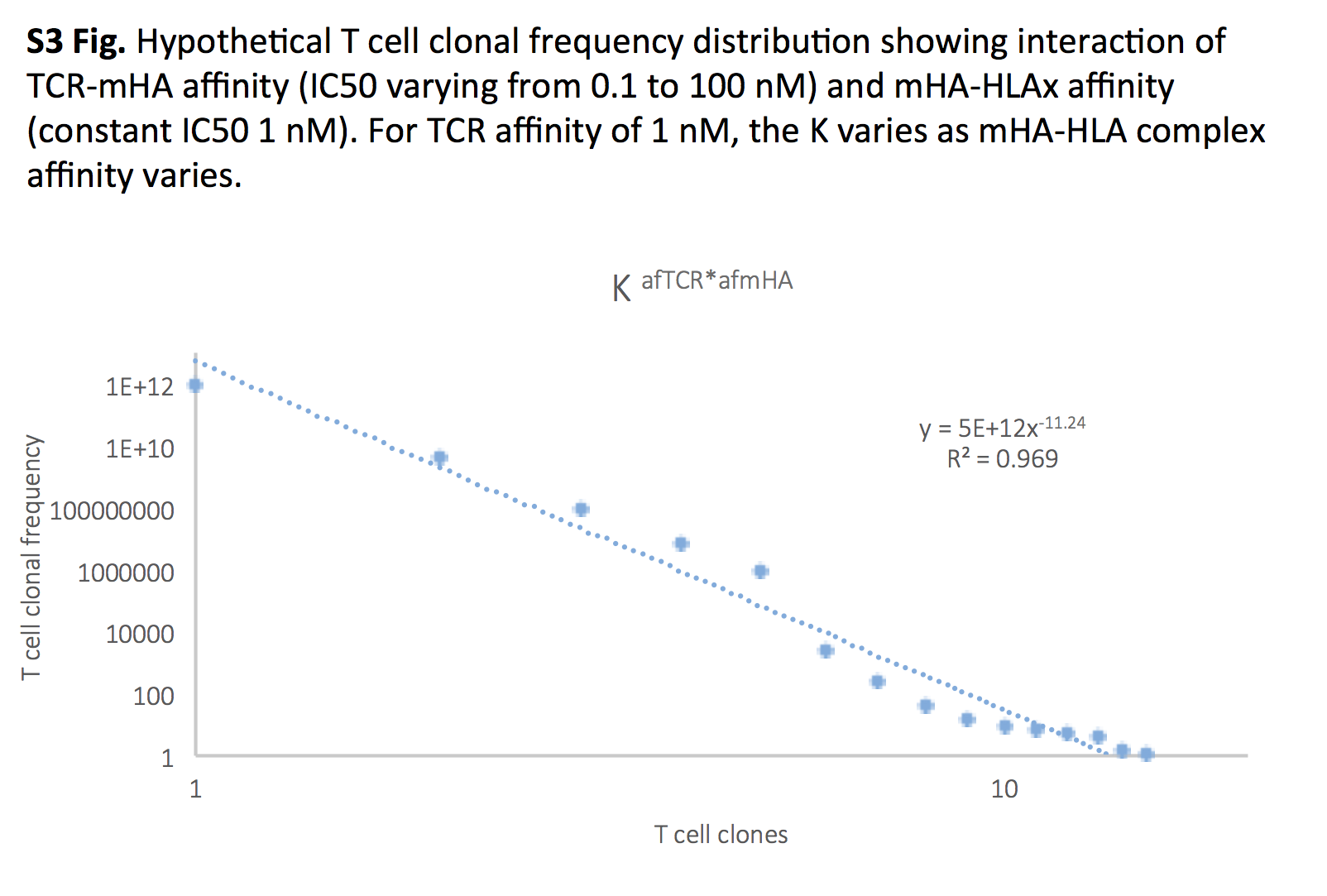

Supplement: S3 Fig — For TCR affinity of 1 nM, the K varies as mHA-HLA complex affinity varies. (TIFF) [file pone.0187771.s003.tiff]

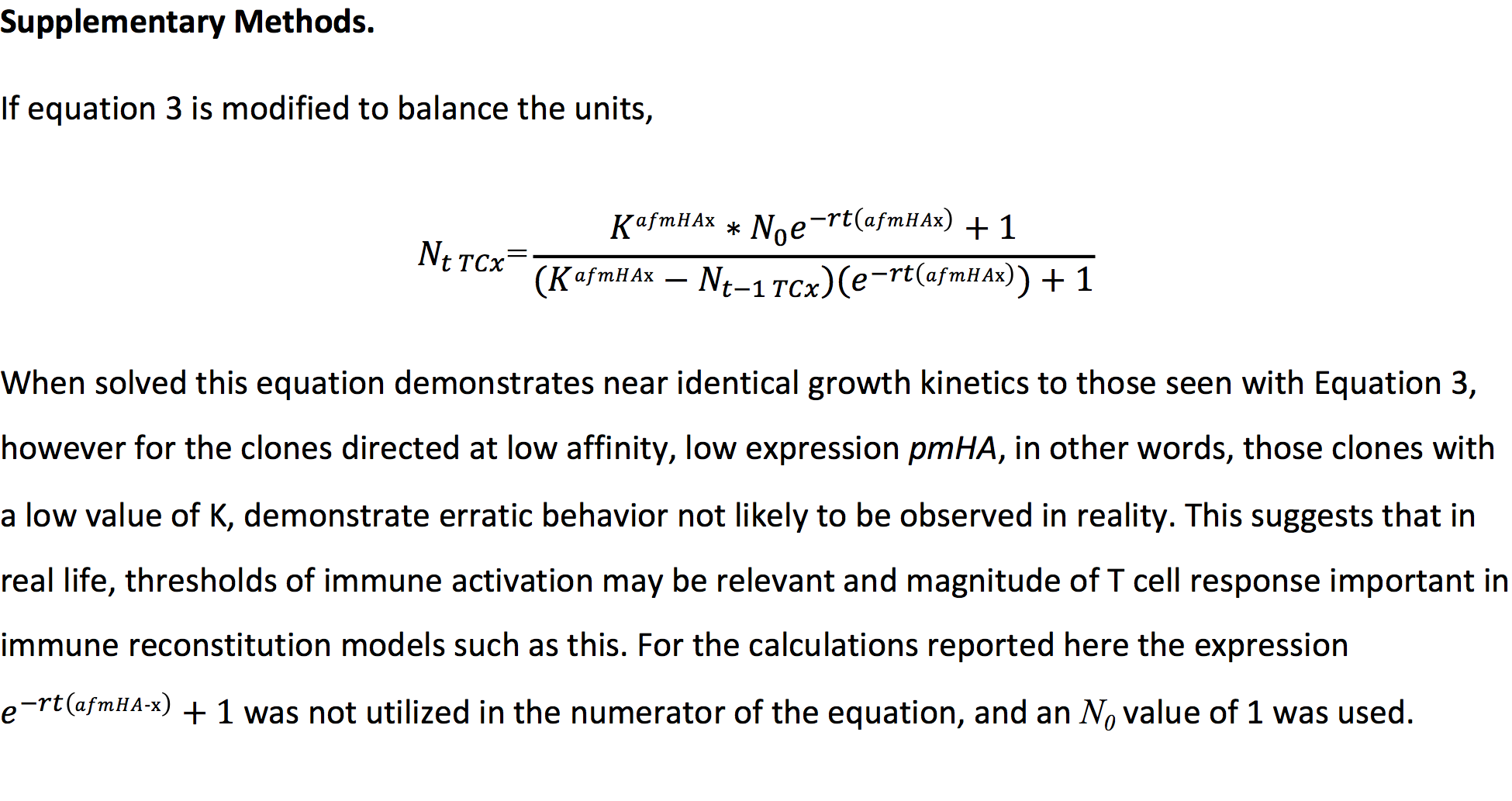

Supplement: S1 Methods — (TIFF) [file pone.0187771.s014.tiff]

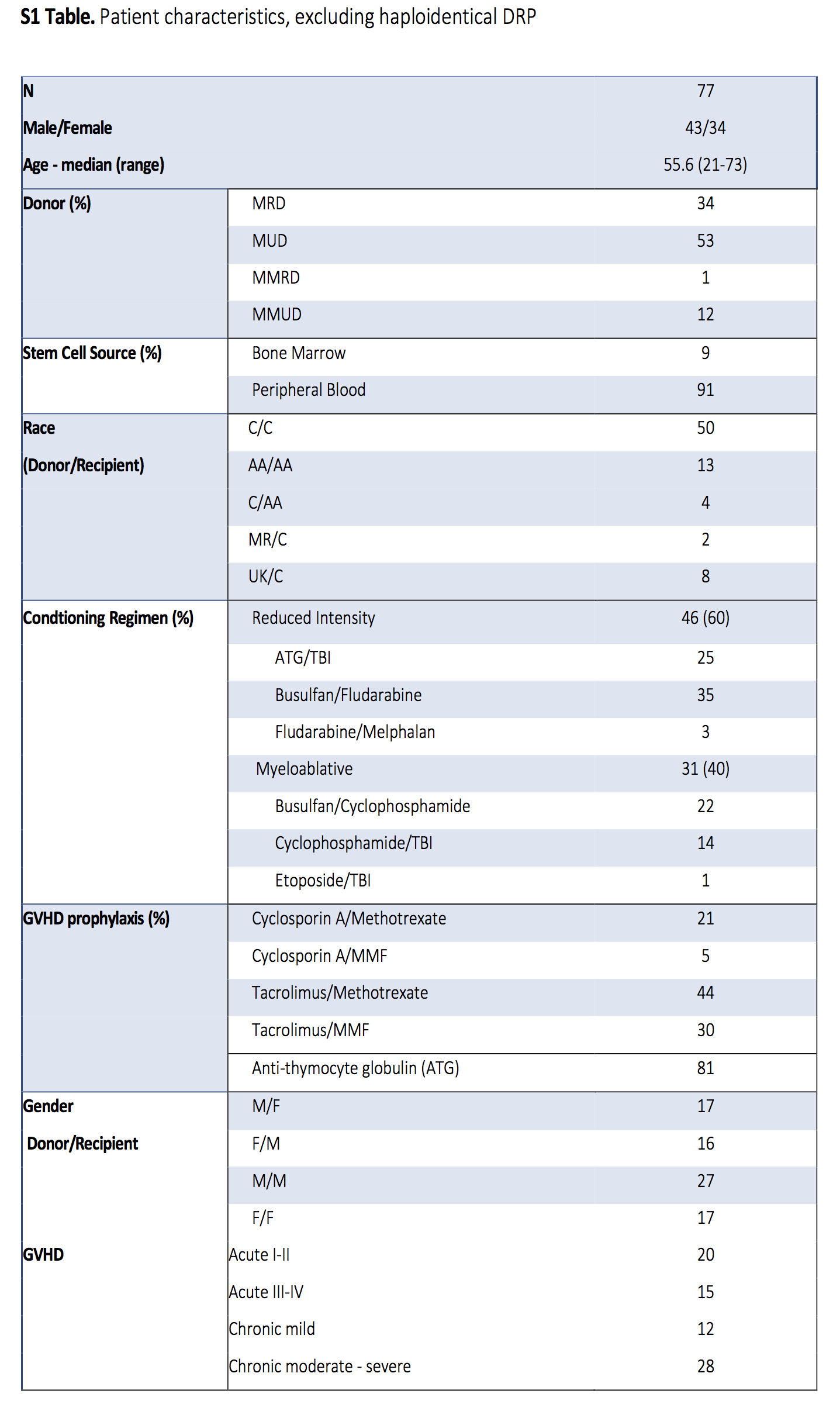

Supplement: S1 Table — (TIFF) [file pone.0187771.s004.tiff]

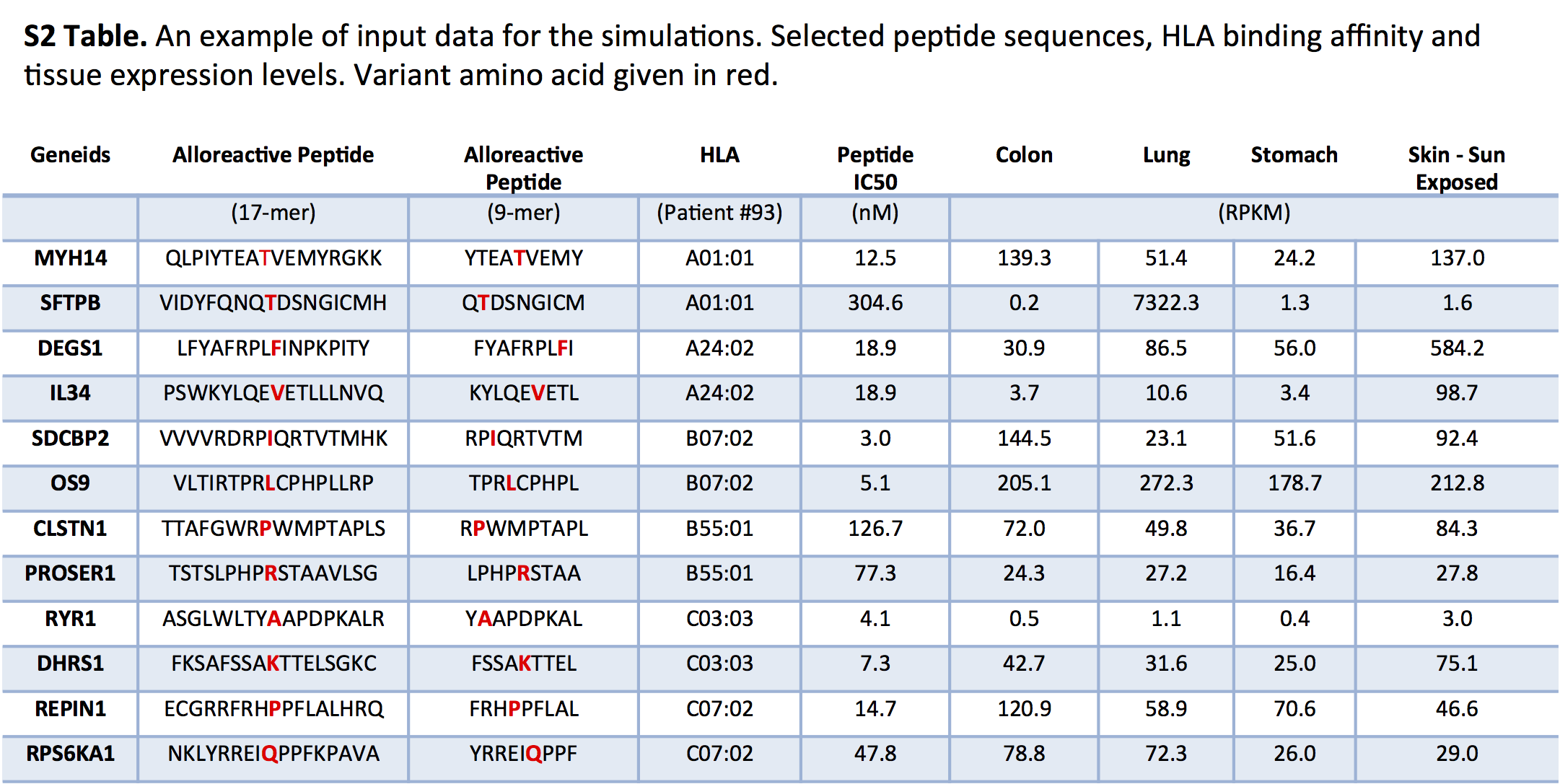

Supplement: S2 Table — Selected peptide sequences, HLA binding affinity and tissue expression levels. Variant amino acid given in red. (TIFF) [file pone.0187771.s005.tiff]

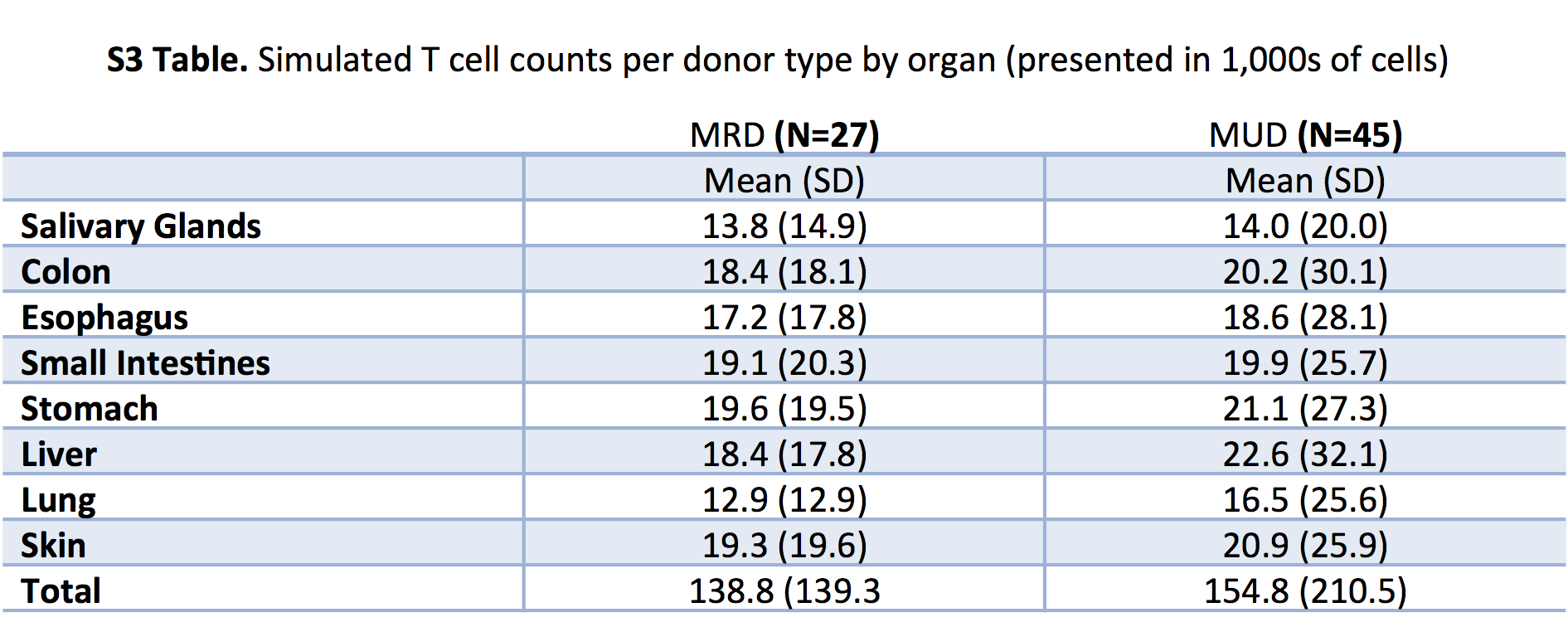

Supplement: S3 Table — (TIFF) [file pone.0187771.s006.tiff]

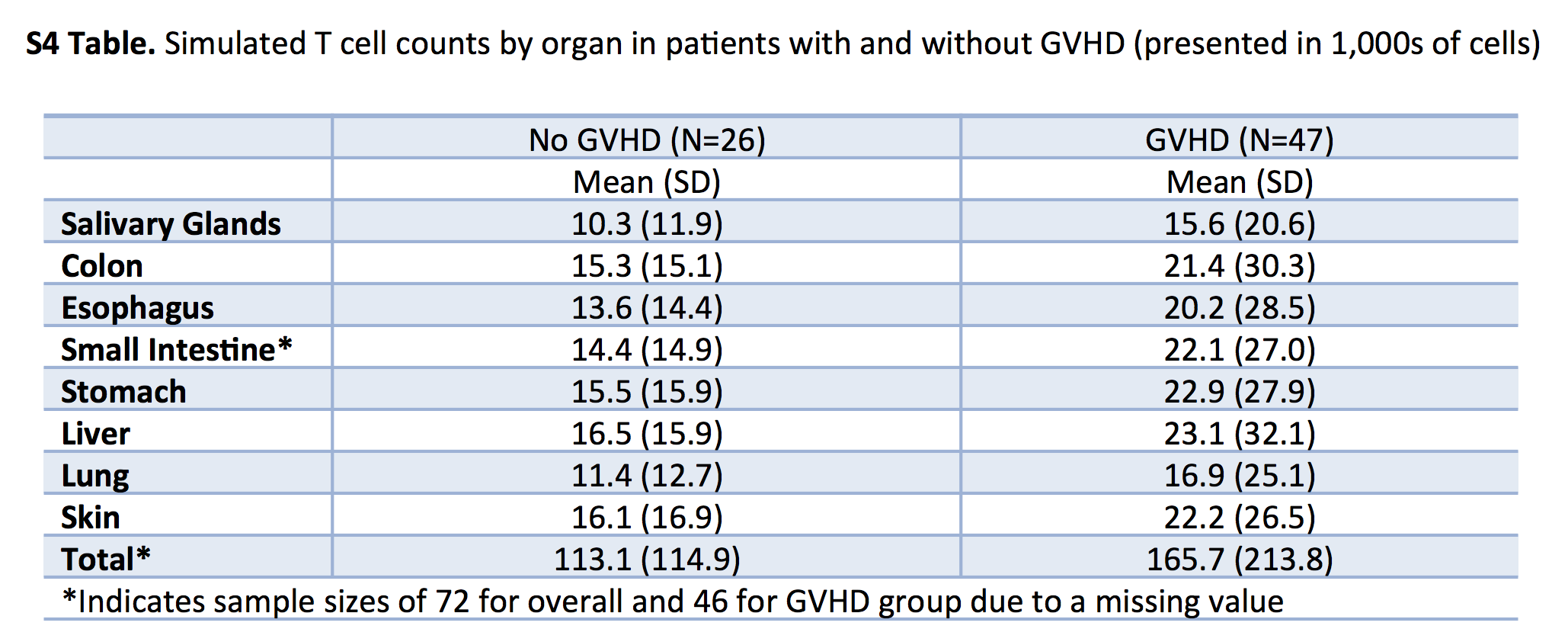

Supplement: S4 Table — (TIFF) [file pone.0187771.s007.tiff]
