# Supplementary material for: Dynamical system modeling to simulate donor T cell response to whole exome sequencing-derived recipient peptides: Understanding randomness in alloreactivity incidence following stem cell transplantation
Source: PLoS One. 2017 Dec 1;12(12):e0187771. doi: 10.1371/journal.pone.0187771 (PMC5711034; doi:10.1371/journal.pone.0187771)
Supplement: S1 File — (DOCX) [file pone.0187771.s008.docx]

This program uses the tissue expression and the IC50 values to simulate the T-cell repertoire post-transplant to determine the alloreactivity of the particular DRP using Eq 3.2. *(The term N_0_ in the numerator was not included in the program, since it is a constant =1)*

clear

tic

%% Reading all files

allfiles = dir;

cellallfiles = struct2cell(allfiles);

cellallfiles(2:5,:) = [];

%%

colallfiles = size(cellallfiles,2);

count = 1;

for y = 1:colallfiles

recog = strfind(cellallfiles(1,y),'xlsx');

compare = cell2mat(recog);

if compare>0

finalfiles(count,1) = cellallfiles(1,y);

count = count +1;

end

end

%% Extracting all the xlsx files from the folder

rowallfiles = size(finalfiles,1);

for y = 1:rowallfiles

finalfiles(y,1) = strrep(finalfiles(y,1), '.xlsx', '');

end

%% Starting simulation algorithm

for compile = 1 : rowallfiles

filename = finalfiles(compile,1);

filenames = strjoin(filename);

[~,~,raw] = xlsread(filenames);

%%enter filename here

%%enter limit of IC50 here

%%number of iterations

reps = 500;

limit = 500;

toc

%%

colsm = size(raw,2);

colcount = 1;

i=1;

%% Reading the expression values

while colcount==1 && i<=colsm

c = strfind(raw(1,i),'Bladder');

d = cell2mat(c);

if d>0

expression(:,colcount) = raw(:,i);

colcount = colcount + 1;

end

i = i+1;

end

while colcount==2 && i<=colsm

c = strfind(raw(1,i),'Blood');

d = cell2mat(c);

if d>0

expression(:,colcount) = raw(:,i);

colcount = colcount + 1;

end

i = i+1;

end

while colcount==3 && i<=colsm

c = strfind(raw(1,i),'Blood Vessel');

d = cell2mat(c);

if d>0

expression(:,colcount) = raw(:,i);

colcount = colcount + 1;

end

i = i+1;

end

while colcount==4 && i<=colsm

c = strfind(raw(1,i),'Colon');

d = cell2mat(c);

if d>0

expression(:,colcount) = raw(:,i);

colcount = colcount + 1;

end

i = i+1;

end

while colcount==5 && i<=colsm

c = strfind(raw(1,i),'Esophagus');

d = cell2mat(c);

if d>0

expression(:,colcount) = raw(:,i);

colcount = colcount + 1;

end

i = i+1;

end

while colcount==6 && i<=colsm

c = strfind(raw(1,i),'Heart');

d = cell2mat(c);

if d>0

expression(:,colcount) = raw(:,i);

colcount = colcount + 1;

end

i = i+1;

end

while colcount==7 && i<=colsm

c = strfind(raw(1,i),'Kidney');

d = cell2mat(c);

if d>0

expression(:,colcount) = raw(:,i);

colcount = colcount + 1;

end

i = i+1;

end

while colcount==8 && i<=colsm

c = strfind(raw(1,i),'Liver');

d = cell2mat(c);

if d>0

expression(:,colcount) = raw(:,i);

colcount = colcount + 1;

end

i = i+1;

end

while colcount==9 && i<=colsm

c = strfind(raw(1,i),'Lung');

d = cell2mat(c);

if d>0

expression(:,colcount) = raw(:,i);

colcount = colcount + 1;

end

i = i+1;

end

while colcount==10 && i<=colsm

c = strfind(raw(1,i),'Muscle');

d = cell2mat(c);

if d>0

expression(:,colcount) = raw(:,i);

colcount = colcount + 1;

end

i = i+1;

end

while colcount==11 && i<=colsm

c = strfind(raw(1,i),'Salivary Gland');

d = cell2mat(c);

if d>0

expression(:,colcount) = raw(:,i);

colcount = colcount + 1;

end

i = i+1;

end

while colcount==12 && i<=colsm

c = strfind(raw(1,i),'Skin');

d = cell2mat(c);

if d>0

expression(:,colcount) = raw(:,i);

colcount = colcount + 1;

end

i = i+1;

end

while colcount==13 && i<=colsm

c = strfind(raw(1,i),'Small Intestine');

d = cell2mat(c);

if d>0

expression(:,colcount) = raw(:,i);

colcount = colcount + 1;

end

i = i+1;

end

while colcount==14 && i<=colsm

c = strfind(raw(1,i),'Spleen');

d = cell2mat(c);

if d>0

expression(:,colcount) = raw(:,i);

colcount = colcount + 1;

end

i = i+1;

end

while colcount==15 && i<=colsm

c = strfind(raw(1,i),'Stomach');

d = cell2mat(c);

if d>0

expression(:,colcount) = raw(:,i);

colcount = colcount + 1;

end

i = i+1;

end

while colcount==16 && i<=colsm

c = strfind(raw(1,i),'Thyroid');

d = cell2mat(c);

if d>0

expression(:,colcount) = raw(:,i);

colcount = colcount + 1;

end

i = i+1;

end

while colcount==17 && i<=colsm

c = strfind(raw(1,i),'Vagina');

d = cell2mat(c);

if d>0

expression(:,colcount) = raw(:,i);

colcount = colcount + 1;

end

i = i+1;

end

%%

colcount = 1;

for i = 1:colsm

c = strfind(raw(1,i),'HLA');

d = cell2mat(c);

if d>0

data(:,colcount) = raw(:,i);

colcount = colcount+1;

end

end

%% Compiling expression and IC50 values

colsm = size(data,2);

z=0;

for i = 1:colsm

c = strfind(data(1,i),'_');

d = cell2mat(c);

if d>0

z=z+1;

end

end

%% Organizing data to optimize the process

i=1;

while z>0

c = strfind(data(1,i),'_');

d = cell2mat(c);

if d>0

data(:,i) = [];

z=z-1;

i=i-1;

end

i=i+1;

end

%% Clearing garbage values

data(1,:)=[];

expression(1,:)=[];

data_c = cell2mat(data);

exp_c = cell2mat(expression);

clear expression data num raw txt;

%% Limiting expression values below threshold

it = size(data_c,1);

it_c = size(exp_c,2);

it_c2 = size(data_c,2);

z=1;

it_t = it*it_c2;

final = zeros(it_t,(it_c+it_c2));

%%

for y = 1:it_c

for i = 1:it

if exp_c(i,y) <= 1

exp_c(i,y) = 0;

end

end

end

%% Consolidating IC50 values

for y=1:it_c2

for i = 1:it

final(z,1) = data_c(i,y);

final(z,2:(1+it_c)) = exp_c(i,:);

z=z+1;

end

end

clear data_c exp_c

%% Limiting IC50 values above threshold

final = sortrows(final);

it_c = size(final,1);

z=0;

%%

z=0;

for i = 1:it_c

if final(i,1)<limit

z=z+1;

end

end

%%

final2 = final(1:z,:);

%% Generating Alpha Matrix

it_c = size(final2,1);

r=1;

a=1;

e=1;

wf=zeros(it_c);

for y = 1:(it_c)

for x = 1:(it_c)

wf(x,y)=final2(r,e)/final2(a,e);

a=a+1;

end

a=1;

r=r+1;

end

%%

final2 (:,1) = 1./final2 (:,1);

ic = final2(:,1);

%% Generating steady state values

k_bladder=1000000.*final2(:,2);

k_blood=1000000.*final2(:,3);

k_bv=1000000.*final2(:,4);

k_colon=1000000.*final2(:,5);

k_e=1000000.*final2(:,6);

k_h=1000000.*final2(:,7);

k_liver=1000000.*final2(:,8);

k_lung=1000000.*final2(:,9);

k_m=1000000.*final2(:,10);

k_sg=1000000.*final2(:,11);

k_skin=1000000.*final2(:,12);

k_si=1000000.*final2(:,13);

k_spleen=1000000.*final2(:,14);

k_stomach=1000000.*final2(:,15);

k_t=1000000.*final2(:,16);

k_v=1000000.*final2(:,17);

toc

%%

z=0;

resultkbv = zeros ((it_c+1),reps);

nt_1sum=zeros((it_c),1);

c=1;

y=1;

resultkbv(:,1)=1;

resultkbv(it_c+1,y) = it_c;

%% Generating Alloreativity potential ignoring expression values below the threshold

a=1;

for y = 1:(reps-1)

for i = 1:it_c

if k_bv(i,1) == 0

nt_1sum(i,1)=0;

else

nt_1 = resultkbv(1:it_c,y);

wfa = wf(1:it_c,i);

nt_1sum(i,1) = dot(nt_1,wfa);

end

end

a=a+1;

sum1 = 0;

for x = 1:it_c

if k_bv(x,1)== 0

resultkbv(x,a) = 0;

else

first = 1;

c=nt_1sum(x,first);

b= ic(x,first);

k = k_bv(x,first)^b;

d = exp(-b*1.5*a);

hla = (k)/((k-c)*d+1);

resultkbv(x,a)=hla;

sum1=sum1+hla;

end

end

resultkbv(it_c+1,a)=sum1;

end

toc

%%

z=0;

resultkbladder = zeros ((it_c+1),reps);

nt_1sum=zeros((it_c),1);

c=1;

y=1;

resultkbladder(:,1)=1;

resultkbladder(it_c+1,y) = it_c;

%%

a=1;

for y = 1:(reps-1)

for i = 1:it_c

if k_bladder(i,1) == 0

nt_1sum(i,1) = 0;

else

nt_1 = resultkbladder(1:it_c,y);

wfa = wf(1:it_c,i);

nt_1sum(i,1) = dot(nt_1,wfa);

end

end

a=a+1;

sum1 = 0;

for x = 1:it_c

if k_bladder(x,1)==0

resultkbladder(x,a) = 0;

else

first = 1;

c=nt_1sum(x,first);

b= ic(x,first);

k = k_bladder(x,first)^b;

d = exp(-b*1.5*a);

hla = (k)/((k-c)*d+1);

resultkbladder(x,a)=hla;

sum1=sum1+hla;

end

end

resultkbladder(it_c+1,a)=sum1;

end

toc

%%

z=0;

resultkcolon = zeros ((it_c+1),reps);

nt_1sum=zeros((it_c),1);

c=1;

y=1;

resultkcolon(:,1)=1;

resultkcolon(it_c+1,y) = it_c;

%%

a=1;

for y = 1:(reps-1)

for i = 1:it_c

if k_colon(i,1)==0

nt_1sum(i,1)=0;

else

nt_1 = resultkcolon(1:it_c,y);

wfa = wf(1:it_c,i);

nt_1sum(i,1) = dot(nt_1,wfa);

end

end

a=a+1;

sum1 = 0;

for x = 1:it_c

if k_colon(x,1) ==0

resultkcolon(x,a) = 0;

else

first = 1;

c=nt_1sum(x,first);

b= ic(x,first);

k = k_colon(x,first)^b;

d = exp(-b*1.5*a);

hla = (k)/((k-c)*d+1);

resultkcolon(x,a)=hla;

sum1=sum1+hla;

end

end

resultkcolon(it_c+1,a)=sum1;

end

toc

%%

z=0;

resultkblood = zeros ((it_c+1),reps);

nt_1sum=zeros((it_c),1);

c=1;

y=1;

resultkblood(:,1)=1;

resultkblood(it_c+1,y) = it_c;

%%

a=1;

for y = 1:(reps-1)

for i = 1:it_c

if k_blood(i,1) == 0

nt_1sum(i,1) = 0;

else

nt_1 = resultkblood(1:it_c,y);

wfa = wf(1:it_c,i);

nt_1sum(i,1) = dot(nt_1,wfa);

end

end

a=a+1;

sum1 = 0;

for x = 1:it_c

if k_blood(x,1) ==0

resultkblood(x,a) = 0;

else

first = 1;

c=nt_1sum(x,first);

b= ic(x,first);

k = k_blood(x,first)^b;

d = exp(-b*1.5*a);

hla = (k)/((k-c)*d+1);

resultkblood(x,a)=hla;

sum1=sum1+hla;

end

end

resultkblood(it_c+1,a)=sum1;

end

toc

%%

z=0;

resultke = zeros ((it_c+1),reps);

nt_1sum=zeros((it_c),1);

c=1;

y=1;

resultke(:,1)=1;

resultke(it_c+1,y) = it_c;

%%

a=1;

for y = 1:(reps-1)

for i = 1:it_c

if k_e(i,1)==0

nt_1sum(i,1) =0;

else

nt_1 = resultke(1:it_c,y);

wfa = wf(1:it_c,i);

nt_1sum(i,1) = dot(nt_1,wfa);

end

end

a=a+1;

sum1 = 0;

for x = 1:it_c

if k_e(x,1) ==0

resultke(x,a) =0;

else

first = 1;

c=nt_1sum(x,first);

b= ic(x,first);

k = k_e(x,first)^b;

d = exp(-b*1.5*a);

hla = (k)/((k-c)*d+1);

resultke(x,a)=hla;

sum1=sum1+hla;

end

end

resultke(it_c+1,a)=sum1;

end

toc

%%

z=0;

resultkh = zeros ((it_c+1),reps);

nt_1sum=zeros((it_c),1);

c=1;

y=1;

resultkh(:,1)=1;

resultkh(it_c+1,y) = it_c;

%%

a=1;

for y = 1:(reps-1)

for i = 1:it_c

if k_h(i,1)==0

nt_1sum(i,1) = 0;

else

nt_1 = resultkh(1:it_c,y);

wfa = wf(1:it_c,i);

nt_1sum(i,1) = dot(nt_1,wfa);

end

end

a=a+1;

sum1 = 0;

for x = 1:it_c

if k_h(x,1) == 0

resultkh(x,a) = 0;

else

first = 1;

c=nt_1sum(x,first);

b= ic(x,first);

k = k_h(x,first)^b;

d = exp(-b*1.5*a);

hla = (k)/((k-c)*d+1);

resultkh(x,a)=hla;

sum1=sum1+hla;

end

end

resultkh(it_c+1,a)=sum1;

end

toc

%%

z=0;

resultkskin = zeros ((it_c+1),reps);

nt_1sum=zeros((it_c),1);

c=1;

y=1;

resultkskin(:,1)=1;

resultkskin(it_c+1,y) = it_c;

%%

a=1;

for y = 1:(reps-1)

for i = 1:it_c

if k_skin(i,1) ==0

nt_1sum(i,1) = 0;

else

nt_1 = resultkskin(1:it_c,y);

wfa = wf(1:it_c,i);

nt_1sum(i,1) = dot(nt_1,wfa);

end

end

a=a+1;

sum1 = 0;

for x = 1:it_c

if k_skin(x,1)==0

resultkskin(x,a)=0;

else

first = 1;

c=nt_1sum(x,first);

b= ic(x,first);

k = k_skin(x,first)^b;

d = exp(-b*1.5*a);

hla = (k)/((k-c)*d+1);

resultkskin(x,a)=hla;

sum1=sum1+hla;

end

end

resultkskin(it_c+1,a)=sum1;

end

toc

%%

z=0;

resultksg = zeros ((it_c+1),reps);

nt_1sum=zeros((it_c),1);

c=1;

y=1;

resultksg(:,1)=1;

resultksg(it_c+1,y) = it_c;

%%

a=1;

for y = 1:(reps-1)

for i = 1:it_c

if k_sg(i,1)==0

nt_1sum(i,1) = 0;

else

nt_1 = resultksg(1:it_c,y);

wfa = wf(1:it_c,i);

nt_1sum(i,1) = dot(nt_1,wfa);

end

end

a=a+1;

sum1 = 0;

for x = 1:it_c

if k_sg(x,1)==0

resultksg(x,a) = 0;

else

first = 1;

c=nt_1sum(x,first);

b= ic(x,first);

k = k_sg(x,first)^b;

d = exp(-b*1.5*a);

hla = (k)/((k-c)*d+1);

resultksg(x,a)=hla;

sum1=sum1+hla;

end

end

resultksg(it_c+1,a)=sum1;

end

toc

%%

z=0;

resultkliver = zeros ((it_c+1),reps);

nt_1sum=zeros((it_c),1);

c=1;

y=1;

resultkliver(:,1)=1;

resultkliver(it_c+1,y) = it_c;

%%

a=1;

for y = 1:(reps-1)

for i = 1:it_c

if k_liver(i,1)==0

nt_1sum(i,1) = 0;

else

nt_1 = resultkliver(1:it_c,y);

wfa = wf(1:it_c,i);

nt_1sum(i,1) = dot(nt_1,wfa);

end

end

a=a+1;

sum1 = 0;

for x = 1:it_c

if k_liver(x,1) ==0

resultkliver(x,a) = 0;

else

first = 1;

c=nt_1sum(x,first);

b= ic(x,first);

k = k_liver(x,first)^b;

d = exp(-b*1.5*a);

hla = (k)/((k-c)*d+1);

resultkliver(x,a)=hla;

sum1=sum1+hla;

end

end

resultkliver(it_c+1,a)=sum1;

end

toc

%%

z=0;

resultkm = zeros ((it_c+1),reps);

nt_1sum=zeros((it_c),1);

c=1;

y=1;

resultkm(:,1)=1;

resultkm(it_c+1,y) = it_c;

%%

a=1;

for y = 1:(reps-1)

for i = 1:it_c

if k_m(i,1)==0

nt_1sum(i,1) = 0;

else

nt_1 = resultkm(1:it_c,y);

wfa = wf(1:it_c,i);

nt_1sum(i,1) = dot(nt_1,wfa);

end

end

a=a+1;

sum1 = 0;

for x = 1:it_c

if k_m(x,1) ==0

resultkm(x,a) = 0;

else

first = 1;

c=nt_1sum(x,first);

b= ic(x,first);

k = k_m(x,first)^b;

d = exp(-b*1.5*a);

hla = (k)/((k-c)*d+1);

resultkm(x,a)=hla;

sum1=sum1+hla;

end

end

resultkm(it_c+1,a)=sum1;

end

toc

%%

z=0;

resultklung = zeros ((it_c+1),reps);

nt_1sum=zeros((it_c),1);

c=1;

y=1;

resultklung(:,1)=1;

resultklung(it_c+1,y) = it_c;

%%

a=1;

for y = 1:(reps-1)

for i = 1:it_c

if k_lung(i,1)==0

nt_1sum(i,1) = 0;

else

nt_1 = resultklung(1:it_c,y);

wfa = wf(1:it_c,i);

nt_1sum(i,1) = dot(nt_1,wfa);

end

end

a=a+1;

sum1 = 0;

for x = 1:it_c

if k_lung(x,1)==0

resultklung(x,a)=0;

else

first = 1;

c=nt_1sum(x,first);

b= ic(x,first);

k = k_lung(x,first)^b;

d = exp(-b*1.5*a);

hla = (k)/((k-c)*d+1);

resultklung(x,a)=hla;

sum1=sum1+hla;

end

end

resultklung(it_c+1,a)=sum1;

end

toc

%%

z=0;

resultksi = zeros ((it_c+1),reps);

nt_1sum=zeros((it_c),1);

c=1;

y=1;

resultksi(:,1)=1;

resultksi(it_c+1,y) = it_c;

%%

a=1;

for y = 1:(reps-1)

for i = 1:it_c

if k_si(i,1)==0;

nt_1sum(i,1)=0;

else

nt_1 = resultksi(1:it_c,y);

wfa = wf(1:it_c,i);

nt_1sum(i,1) = dot(nt_1,wfa);

end

end

a=a+1;

sum1 = 0;

for x = 1:it_c

if k_si(x,1) == 0

resultksi(x,a) = 0;

else

first = 1;

c=nt_1sum(x,first);

b= ic(x,first);

k = k_si(x,first)^b;

d = exp(-b*1.5*a);

hla = (k)/((k-c)*d+1);

resultksi(x,a)=hla;

sum1=sum1+hla;

end

end

resultksi(it_c+1,a)=sum1;

end

toc

%%

z=0;

resultkspleen = zeros ((it_c+1),reps);

nt_1sum=zeros((it_c),1);

c=1;

y=1;

resultkspleen(:,1)=1;

resultkspleen(it_c+1,y) = it_c;

%%

a=1;

for y = 1:(reps-1)

for i = 1:it_c

if k_spleen(i,1)==0

nt_1sum(i,1)=0;

else

nt_1 = resultkspleen(1:it_c,y);

wfa = wf(1:it_c,i);

nt_1sum(i,1) = dot(nt_1,wfa);

end

end

a=a+1;

sum1 = 0;

for x = 1:it_c

if k_spleen(x,1)==0;

resultkspleen(x,a)=0;

else

first = 1;

c=nt_1sum(x,first);

b= ic(x,first);

k = k_spleen(x,first)^b;

d = exp(-b*1.5*a);

hla = (k)/((k-c)*d+1);

resultkspleen(x,a)=hla;

sum1=sum1+hla;

end

end

resultkspleen(it_c+1,a)=sum1;

end

toc

%%

z=0;

resultkstomach = zeros ((it_c+1),reps);

nt_1sum=zeros((it_c),1);

c=1;

y=1;

resultkstomach(:,1)=1;

resultkstomach(it_c+1,y) = it_c;

%%

a=1;

for y = 1:(reps-1)

for i = 1:it_c

if k_stomach(i,1)==0

nt_1sum(i,1)=0;

else

nt_1 = resultkstomach(1:it_c,y);

wfa = wf(1:it_c,i);

nt_1sum(i,1) = dot(nt_1,wfa);

end

end

a=a+1;

sum1 = 0;

for x = 1:it_c

if k_stomach(x,1)==0

resultkstomach(x,a)=0;

else

first = 1;

c=nt_1sum(x,first);

b= ic(x,first);

k = k_stomach(x,first)^b;

d = exp(-b*1.5*a);

hla = (k)/((k-c)*d+1);

resultkstomach(x,a)=hla;

sum1=sum1+hla;

end

end

resultkstomach(it_c+1,a)=sum1;

end

toc

%%

z=0;

resultkt = zeros ((it_c+1),reps);

nt_1sum=zeros((it_c),1);

c=1;

y=1;

resultkt(:,1)=1;

resultkt(it_c+1,y) = it_c;

%%

a=1;

for y = 1:(reps-1)

for i = 1:it_c

if k_t(i,1)==0

nt_1sum(i,1)=0;

else

nt_1 = resultkt(1:it_c,y);

wfa = wf(1:it_c,i);

nt_1sum(i,1) = dot(nt_1,wfa);

end

end

a=a+1;

sum1 = 0;

for x = 1:it_c

if k_t(x,1)==0

resultkt(x,a)=0;

else

first = 1;

c=nt_1sum(x,first);

b= ic(x,first);

k = k_t(x,first)^b;

d = exp(-b*1.5*a);

hla = (k)/((k-c)*d+1);

resultkt(x,a)=hla;

sum1=sum1+hla;

end

end

resultkt(it_c+1,a)=sum1;

end

toc

%%

z=0;

resultkv = zeros ((it_c+1),reps);

nt_1sum=zeros((it_c),1);

c=1;

y=1;

resultkv(:,1)=1;

resultkv(it_c+1,y) = it_c;

%%

a=1;

for y = 1:(reps-1)

for i = 1:it_c

if k_v(i,1)==0

nt_1sum(i,1)=0;

else

nt_1 = resultkv(1:it_c,y);

wfa = wf(1:it_c,i);

nt_1sum(i,1) = dot(nt_1,wfa);

end

end

a=a+1;

sum1 = 0;

for x = 1:it_c

if k_v(x,1)==0

resultkv(x,a) = 0;

else

first = 1;

c=nt_1sum(x,first);

b= ic(x,first);

k = k_v(x,first)^b;

d = exp(-b*1.5*a);

hla = (k)/((k-c)*d+1);

resultkv(x,a)=hla;

sum1=sum1+hla;

end

end

resultkv(it_c+1,a)=sum1;

end

toc

%% calculating averages for the last 100 iterations of sum of all clones

average = cell(16,2);

bv = resultkbv((it_c+1),401:500);

avgbv = sum(bv)/100;

average(1,1) = {'blood vessel'};

average(1,2) = {avgbv};

bv = resultkbladder((it_c+1),401:500);

avgbv = sum(bv)/100;

average(2,1) = {'bladder'};

average(2,2) = {avgbv};

bv = resultkblood((it_c+1),401:500);

avgbv = sum(bv)/100;

average(3,1) = {'blood'};

average(3,2) = {avgbv};

bv = resultkcolon((it_c+1),401:500);

avgbv = sum(bv)/100;

average(4,1) = {'colon'};

average(4,2) = {avgbv};

bv = resultke((it_c+1),401:500);

avgbv = sum(bv)/100;

average(5,1) = {'esophagus'};

average(5,2) = {avgbv};

bv = resultkh((it_c+1),401:500);

avgbv = sum(bv)/100;

average(6,1) = {'heart'};

average(6,2) = {avgbv};

bv = resultkliver((it_c+1),401:500);

avgbv = sum(bv)/100;

average(7,1) = {'liver'};

average(7,2) = {avgbv};

bv = resultklung((it_c+1),401:500);

avgbv = sum(bv)/100;

average(8,1) = {'lung'};

average(8,2) = {avgbv};

bv = resultkm((it_c+1),401:500);

avgbv = sum(bv)/100;

average(9,1) = {'m'};

average(9,2) = {avgbv};

bv = resultksg((it_c+1),401:500);

avgbv = sum(bv)/100;

average(10,1) = {'salivary gland'};

average(10,2) = {avgbv};

bv = resultksi((it_c+1),401:500);

avgbv = sum(bv)/100;

average(11,1) = {'small intestine'};

average(11,2) = {avgbv};

bv = resultkskin((it_c+1),401:500);

avgbv = sum(bv)/100;

average(12,1) = {'skin'};

average(12,2) = {avgbv};

bv = resultkspleen((it_c+1),401:500);

avgbv = sum(bv)/100;

average(13,1) = {'spleen'};

average(13,2) = {avgbv};

bv = resultkstomach((it_c+1),401:500);

avgbv = sum(bv)/100;

average(14,1) = {'stomach'};

average(14,2) = {avgbv};

bv = resultkt((it_c+1),401:500);

avgbv = sum(bv)/100;

average(15,1) = {'t'};

average(15,2) = {avgbv};

bv = resultkv((it_c+1),401:500);

avgbv = sum(bv)/100;

average(16,1) = {'v'};

average(16,2) = {avgbv};

%% Consolidating alloreactivity potential for all organs

result_f = resultkbv+resultkcolon+resultkbladder+resultkblood+resultke+resultkh+resultkskin+resultksg+resultkliver+resultkm+resultklung+resultksi+resultkspleen+resultkstomach+resultkt+resultkv;

%% Generating files for individual organs and sum of all organs

file = 'mf.xlsx';

files = strjoin(filename,file);

files = strcat(files,file);

xlswrite(files,result_f)

file = 'bv.xlsx';

files = strjoin(filename,file);

files = strcat(files,file);

xlswrite(files,resultkbv)

file = 'colon.xlsx';

files = strjoin(filename,file);

files = strcat(files,file);

xlswrite(files,resultkcolon)

file = 'bladder.xlsx';

files = strjoin(filename,file);

files = strcat(files,file);

xlswrite(files,resultkbladder)

file = 'blood.xlsx';

files = strjoin(filename,file);

files = strcat(files,file);

xlswrite(files,resultkblood)

file = 'e.xlsx';

files = strjoin(filename,file);

files = strcat(files,file);

xlswrite(files,resultke)

file = 'h.xlsx';

files = strjoin(filename,file);

files = strcat(files,file);

xlswrite(files,resultkh)

file = 'skin.xlsx';

files = strjoin(filename,file);

files = strcat(files,file);

xlswrite(files,resultkskin)

file = 'sg.xlsx';

files = strjoin(filename,file);

files = strcat(files,file);

xlswrite(files,resultksg)

file = 'liver.xlsx';

files = strjoin(filename,file);

files = strcat(files,file);

xlswrite(files,resultkliver)

file = 'm.xlsx';

files = strjoin(filename,file);

files = strcat(files,file);

xlswrite(files,resultkm)

file = 'lung.xlsx';

files = strjoin(filename,file);

files = strcat(files,file);

xlswrite(files,resultklung)

file = 'si.xlsx';

files = strjoin(filename,file);

files = strcat(files,file);

xlswrite(files,resultksi)

file = 'mf6.xlsx';

files = strjoin(filename,file);

files = strcat(files,file);

xlswrite(files,result_f6)

file = 'spleen.xlsx';

files = strjoin(filename,file);

files = strcat(files,file);

xlswrite(files,resultkspleen)

file = 'stomach.xlsx';

files = strjoin(filename,file);

files = strcat(files,file);

xlswrite(files,resultkstomach)

file = 't.xlsx';

files = strjoin(filename,file);

files = strcat(files,file);

xlswrite(files,resultkt)

file = 'v.xlsx';

files = strjoin(filename,file);

files = strcat(files,file);

xlswrite(files,resultkv)

file = 'avg.xlsx';

files = strjoin(filename,file);

files = strcat(files,file);

xlswrite(files,average)

toc

end
