# Supplementary material for: Dynamical system modeling to simulate donor T cell response to whole exome sequencing-derived recipient peptides: Understanding randomness in alloreactivity incidence following stem cell transplantation
Source: PLoS One. 2017 Dec 1;12(12):e0187771. doi: 10.1371/journal.pone.0187771 (PMC5711034; doi:10.1371/journal.pone.0187771)
Supplement: S2 File — (DOCX) [file pone.0187771.s009.docx]

This program generates tissue expression for all the peptides across the miHA-HLA complex for all HLA types

clear

tic

%% Reading the DRP files

allfiles = dir;

cellallfiles = struct2cell(allfiles);

cellallfiles(2:5,:) = [];

%%

colallfiles = size(cellallfiles,2);

count = 1;

for y = 1:colallfiles

recog = strfind(cellallfiles(1,y),'xlsx');

compare = cell2mat(recog);

if compare>0

finalfiles(count,1) = cellallfiles(1,y);

count = count +1;

end

end

%%

rowallfiles = size(finalfiles,1);

for y = 1:rowallfiles

finalfiles(y,1) = strrep(finalfiles(y,1), '.xlsx', '');

end

%% Initiating tissue identification

t_dis = cell(79,18);

t_dis(1,1) = cellstr('Patient');

t_dis(1,2) = cellstr('Bladder');

t_dis(1,3) = cellstr('Blood');

t_dis(1,4) = cellstr('Blood Vessel');

t_dis(1,5) = cellstr('Colon');

t_dis(1,6) = cellstr('Esophagus');

t_dis(1,7) = cellstr('Heart');

t_dis(1,8) = cellstr('Kidney');

t_dis(1,9) = cellstr('Liver');

t_dis(1,10) = cellstr('Lung');

t_dis(1,11) = cellstr('Muscle');

t_dis(1,12) = cellstr('Salivary Gland');

t_dis(1,13) = cellstr('Skin');

t_dis(1,14) = cellstr('Small Intestine');

t_dis(1,15) = cellstr('Spleen');

t_dis(1,16) = cellstr('Stomach');

t_dis(1,17) = cellstr('Thyroid');

t_dis(1,18) = cellstr('Vagina');

%%

for compile = 1 : rowallfiles

filename = finalfiles(compile,1);

filenames = strjoin(filename);

[~,~,raw] = xlsread(filenames);

t_dis((compile+1),1) = filename;

%enter filename here

%enter limit of IC50 here

%number of iterations

limit = 500;

toc

%%

colsm = size(raw,2);

colcount = 1;

i=1;

%%

while colcount==1 && i<=colsm

c = strfind(raw(1,i),'Bladder');

d = cell2mat(c);

if d>0

expression(:,colcount) = raw(:,i);

colcount = colcount + 1;

end

i = i+1;

end

while colcount==2 && i<=colsm

c = strfind(raw(1,i),'Blood');

d = cell2mat(c);

if d>0

expression(:,colcount) = raw(:,i);

colcount = colcount + 1;

end

i = i+1;

end

while colcount==3 && i<=colsm

c = strfind(raw(1,i),'Blood Vessel');

d = cell2mat(c);

if d>0

expression(:,colcount) = raw(:,i);

colcount = colcount + 1;

end

i = i+1;

end

while colcount==4 && i<=colsm

c = strfind(raw(1,i),'Colon');

d = cell2mat(c);

if d>0

expression(:,colcount) = raw(:,i);

colcount = colcount + 1;

end

i = i+1;

end

while colcount==5 && i<=colsm

c = strfind(raw(1,i),'Esophagus');

d = cell2mat(c);

if d>0

expression(:,colcount) = raw(:,i);

colcount = colcount + 1;

end

i = i+1;

end

while colcount==6 && i<=colsm

c = strfind(raw(1,i),'Heart');

d = cell2mat(c);

if d>0

expression(:,colcount) = raw(:,i);

colcount = colcount + 1;

end

i = i+1;

end

while colcount==7 && i<=colsm

c = strfind(raw(1,i),'Kidney');

d = cell2mat(c);

if d>0

expression(:,colcount) = raw(:,i);

colcount = colcount + 1;

end

i = i+1;

end

while colcount==8 && i<=colsm

c = strfind(raw(1,i),'Liver');

d = cell2mat(c);

if d>0

expression(:,colcount) = raw(:,i);

colcount = colcount + 1;

end

i = i+1;

end

while colcount==9 && i<=colsm

c = strfind(raw(1,i),'Lung');

d = cell2mat(c);

if d>0

expression(:,colcount) = raw(:,i);

colcount = colcount + 1;

end

i = i+1;

end

while colcount==10 && i<=colsm

c = strfind(raw(1,i),'Muscle');

d = cell2mat(c);

if d>0

expression(:,colcount) = raw(:,i);

colcount = colcount + 1;

end

i = i+1;

end

while colcount==11 && i<=colsm

c = strfind(raw(1,i),'Salivary Gland');

d = cell2mat(c);

if d>0

expression(:,colcount) = raw(:,i);

colcount = colcount + 1;

end

i = i+1;

end

while colcount==12 && i<=colsm

c = strfind(raw(1,i),'Skin');

d = cell2mat(c);

if d>0

expression(:,colcount) = raw(:,i);

colcount = colcount + 1;

end

i = i+1;

end

while colcount==13 && i<=colsm

c = strfind(raw(1,i),'Small Intestine');

d = cell2mat(c);

if d>0

expression(:,colcount) = raw(:,i);

colcount = colcount + 1;

end

i = i+1;

end

while colcount==14 && i<=colsm

c = strfind(raw(1,i),'Spleen');

d = cell2mat(c);

if d>0

expression(:,colcount) = raw(:,i);

colcount = colcount + 1;

end

i = i+1;

end

while colcount==15 && i<=colsm

c = strfind(raw(1,i),'Stomach');

d = cell2mat(c);

if d>0

expression(:,colcount) = raw(:,i);

colcount = colcount + 1;

end

i = i+1;

end

while colcount==16 && i<=colsm

c = strfind(raw(1,i),'Thyroid');

d = cell2mat(c);

if d>0

expression(:,colcount) = raw(:,i);

colcount = colcount + 1;

end

i = i+1;

end

while colcount==17 && i<=colsm

c = strfind(raw(1,i),'Vagina');

d = cell2mat(c);

if d>0

expression(:,colcount) = raw(:,i);

colcount = colcount + 1;

end

i = i+1;

end

%% Optimizing data structures

colcount = 1;

for i = 1:colsm

c = strfind(raw(1,i),'HLA');

d = cell2mat(c);

if d>0

data(:,colcount) = raw(:,i);

colcount = colcount+1;

end

end

%%

colsm = size(data,2);

z=0;

for i = 1:colsm

c = strfind(data(1,i),'_');

d = cell2mat(c);

if d>0

z=z+1;

end

end

%%

i=1;

while z>0

c = strfind(data(1,i),'_');

d = cell2mat(c);

if d>0

data(:,i) = [];

z=z-1;

i=i-1;

end

i=i+1;

end

%% removing garbage data to optimize the process

data(1,:)=[];

expression(1,:)=[];

data_c = cell2mat(data);

exp_c = cell2mat(expression);

clear data expression

%%

it = size(data_c,1);

it_c = size(exp_c,2);

it_c2 = size(data_c,2);

z=1;

it_t = it*it_c2;

final = zeros(it_t,(it_c+1));

%%

for y=1:it_c2

for i = 1:it

final(z,1) = data_c(i,y);

final(z,2:(1+it_c)) = exp_c(i,:);

z=z+1;

end

end

clear data_c exp_c

%%

final = sortrows(final);

it_c = size(final,1);

z=0;

%%

z=0;

for i = 1:it_c

if final(i,1)<limit

z=z+1;

end

end

%% Initiating distribution algorithm

exp = final(1:z,:);

exp(:,1) = [];

clear final

it_c = size(exp,2);

it_r = size(exp,1);

num = 0;

%%

for y = 1:it_c

num = 0;

for x = 1:it_r

if exp(x,y)>=1

num = num+1;

end

num_cell = num2cell(num);

t_dis((compile+1),(y+1)) = num_cell;

end

end

clear exp

end
