# Supplementary material for: Dynamical system modeling to simulate donor T cell response to whole exome sequencing-derived recipient peptides: Understanding randomness in alloreactivity incidence following stem cell transplantation
Source: PLoS One. 2017 Dec 1;12(12):e0187771. doi: 10.1371/journal.pone.0187771 (PMC5711034; doi:10.1371/journal.pone.0187771)
Supplement: S3 File — (DOCX) [file pone.0187771.s010.docx]

This program reads the HLA type of each peptide. It then generates the HLA specific count for miHA for a particular DRP

clear

tic

%% Reading all the files in a folder

allfiles = dir;

cellallfiles = struct2cell(allfiles);

cellallfiles(2:5,:) = [];

%%

colallfiles = size(cellallfiles,2);

count = 1;

for y = 1:colallfiles

recog = strfind(cellallfiles(1,y),'xlsx');

compare = cell2mat(recog);

if compare>0

finalfiles(count,1) = cellallfiles(1,y);

count = count +1;

end

end

%%

rowallfiles = size(finalfiles,1);

for y = 1:rowallfiles

finalfiles(y,1) = strrep(finalfiles(y,1), '.xlsx', '');

end

%% optimizing data structures

count = cell(78,4);

for compile = 1 : rowallfiles

clear data count_a count_b count_c ic count_data

filename = finalfiles(compile,1);

filenames = strjoin(filename);

[~,num,raw] = xlsread(filenames);

count(compile,1) = filename;

colsm = size(raw,2);

%%

colcount = 1;

for i = 1:colsm

c = strfind(raw(1,i),'HLA');

d = cell2mat(c);

if d>0

data(:,colcount) = raw(:,i);

colcount = colcount+1;

end

end

%%

colsm = size(data,2);

z=0;

for i = 1:colsm

c = strfind(data(1,i),'_');

d = cell2mat(c);

if d>0

z=z+1;

end

end

%%

i=1;

while z>0

c = strfind(data(1,i),'_');

d = cell2mat(c);

if d>0

data(:,i) = [];

z=z-1;

i=i-1;

end

i=i+1;

end

%% Initiating algorithm to identify HLA type for each peptide

it_c = size(data,2);

it_r = size(data,1);

count_data = data(2:it_r,:);

count_data = cell2mat(count_data);

count_data = sort(count_data,'ascend');

count_a = 0;

count_b = 0;

count_c = 0;

%%

for i = 1:it_c

[token,remain] = strtok(data(1,i),'-');

data(1,i) = remain;

%%

type_a = strfind(data(1,i),'A');

type_b = strfind(data(1,i),'B');

type_c = strfind(data(1,i),'C');

type_a = cell2mat(type_a);

type_b = cell2mat(type_b);

type_c = cell2mat(type_c);

%%

if type_a>0

x = 0;

a = 1;

ic = 0;

while ic<=500

ic = count_data(a,i);

a = a+1;

x = x+1;

end

count_a = count_a +x;

cell_a = num2cell(count_a);

count(compile,2) = cell_a;

elseif type_b>0

a = 1;

x = 0;

ic = 0;

while ic<=500

ic = count_data(a,i);

x = x+1;

a = a+1;

end

count_b = count_b +x;

cell_b = num2cell(count_b);

count(compile,3) = cell_b;

else

x = 0;

a = 1;

ic = 0;

while ic<=500

ic = count_data(a,i);

x = x+1;

a = a+1;

end

count_c = count_c +x;

cell_c = num2cell(count_c);

count(compile,4) = cell_c;

end

end

end
