# Supplementary material for: Dynamical system modeling to simulate donor T cell response to whole exome sequencing-derived recipient peptides: Understanding randomness in alloreactivity incidence following stem cell transplantation
Source: PLoS One. 2017 Dec 1;12(12):e0187771. doi: 10.1371/journal.pone.0187771 (PMC5711034; doi:10.1371/journal.pone.0187771)
Supplement: S4 File — (DOCX) [file pone.0187771.s011.docx]

%% This program identifies the HLA type of all the peptides within the Y chromosome.

%% Reading the master file.

clear

[~,~,raw] = xlsread('new y');

it_c = size(raw,2);

it_r = size(raw,1);

drp = raw(2:it_r,1);

drp = cell2mat(drp);

for i = 1:it_c

c = strfind(raw(1,i),'HLA');

d = cell2mat(c);

if d>0

hla_type = raw(:,i);

end

end

%% Optimizing data structures

hla_type(1,:) = [];

it_r = size(drp,1);

for i = 1:it_r

[token,remain] = strtok(hla_type(i,1),'-');

hla_type(i,1) = remain;

end

y_hla = cell(78,4);

x = 1;

y = 1;

%% Initializing HLA recognition algorithm

while x<=it_r

count_a = 0;

count_b = 0;

count_c = 0;

num = drp(x,1);

y_hla(y,1) = num2cell(num);

while x<=it_r && drp(x,1) == num

type_a = strfind(hla_type(x,1),'A');

type_b = strfind(hla_type(x,1),'B');

type_c = strfind(hla_type(x,1),'C');

type_a = cell2mat(type_a);

type_b = cell2mat(type_b);

type_c = cell2mat(type_c);

if type_a>0

count_a = count_a+1;

elseif type_b>0

count_b = count_b+1;

else

count_c = count_c+1;

end

x = x+1;

end

y_hla(y,1) = num2cell(num);

y_hla(y,2) = num2cell(count_a);

y_hla(y,3) = num2cell(count_b);

y_hla(y,4) = num2cell(count_c);

y = y+1;

end
